# In-situ spatial and temporal electrical characterization of ZnO thin films deposited by atmospheric pressure chemical vapour deposition on flexible polymer substrates

**DOI:** 10.1038/s41598-020-76993-4

**Published:** 2020-11-17

**Authors:** Alexander Jones, Kissan Mistry, Manfred Kao, Ahmed Shahin, Mustafa Yavuz, Kevin P. Musselman

**Affiliations:** 1 Department of Mechanical and Mechatronics Engineering, University of Waterloo, Waterloo, ON Canada; 2 Waterloo Institute for Nanotechnology, University of Waterloo, Waterloo, ON Canada

**Keywords:** Characterization and analytical techniques, Electronic devices, Electronic properties and materials

## Abstract

A technique is presented for collecting data on both the spatial and temporal variations in the electrical properties of a film as it is deposited on a flexible substrate. A flexible printed circuit board substrate with parallel electrodes distributed across its surface was designed. Zinc oxide films were then deposited on the flexible substrate at different temperatures via atmospheric pressure chemical vapour deposition (AP-CVD) using a spatial atomic layer deposition system. AP-CVD is a promising high-throughput thin film deposition technique with applications in flexible electronics. Collecting data on the film properties in-situ allows us to directly observe how deposition conditions affect the evolution of those properties in real-time. The spatial uniformity of the growing film was monitored, and the various stages of film nucleation and growth on the polymer substrate were observed. The measured resistance of the films was observed to be very high until a critical amount of material has been deposited, consistent with Volmer–Weber growth. Furthermore, monitoring the film resistance during post-deposition cooling enabled immediate identification of metallic or semiconducting behaviour within the conductive ZnO films. This technique allows for a more complete understanding of metal chalcogen film growth and properties, and the high volume of data generated will be useful for future implementations of machine-learning directed materials science.

## Introduction

Techniques such as chemical vapour deposition^1^ and atomic layer deposition^2^ (CVD, ALD) are used to create thin films and nanoparticles for use in many nanoelectronic devices, such as integrated circuits^3^, photovoltaics^4,5^, gas sensors^6^, display technologies^7^, and flexible electronics^8,9^. Film properties can change throughout a deposition procedure and can also vary across the film either due to growth conditions^10,11^ or as a deliberately induced gradient^12^. Measuring the temporal variation of film properties during deposition can enable characterization of the underlying growth mechanisms and identification and correction of any non-idealities in the deposition process. Measuring the spatial variation can enable characterization of the deposition process. Both are important for designing better films and more efficient devices.

The electrical properties of films are particularly important for wearable and wireless nanoelectronic devices, used in the Internet of Things (IoT) for real-time monitoring. Resistance measurements of thin films have previously been taken in-situ, to study their phase changes^13^, degradation^14^, and formation^15–17^. For example, it has been shown that the resistance of a thin film can change dramatically as the film growth conditions begin to favour epitaxial growth over island formation, and this transition point is strongly influenced by the deposition temperature^16,17^.

In these previous studies, the in-situ measurements^13–17^ were all performed using a four-point probe system to obtain the resistance of the entire film as it changes over time. Hence these measurements provide no information about the spatial variation of film properties. Ex-situ measurements, performed after film fabrication is complete, are required to characterize any spatial variation^18^. Techniques that provide both temporal and spatial information about a film’s resistance during growth would be very valuable. Notably, such techniques would enable larger data sets of measured film properties to be generated, which will be essential for the implementation of machine learning in combinatorial materials science^19,20^.

Additionally, these previous in-situ measurements were done for films deposited by conventional CVD, sputtering, or ALD on traditional rigid substrates. The study of film formation and monitoring of film uniformity for emerging nanomanufacturing techniques and flexible polymer substrates will be critical for the development of next generation electronics. Atmospheric pressure spatial atomic layer deposition and chemical vapour deposition (AP-SALD, AP-CVD), for example, are rapid, scalable, open-air techniques for the deposition of pin-hole free nanoscale films^21–24^. These techniques have been employed in the development of next-generation photovoltaics^25,26^, LEDs^27,28^, transistors^29^, and diodes^30^ and are compatible with deposition on plastic substrates for flexible electronics. Film formation and growth may be fundamentally different on flexible substrates. Metal oxide films produced by ALD grow best on other oxides, due to their surface hydroxyl groups, and nucleation can be a challenge on polymer surfaces. Polymers tend to be permeable, and have few nucleation sites, so the initial few cycles of ALD growth may be slow as the reactants need to permeate within the film first, to allow the subsequent ALD cycles to nucleate on areas with high precursor concentrations within the polymer^31,32^.

Here we present a technique for obtaining non-invasive temporal and spatial electrical measurements on a flexible polymer substrate as a film is deposited by AP-CVD using an AP-SALD system. Since the system operates in open atmosphere, we were able to introduce in-situ electrical measurements to the AP-SALD system using a specially designed printed circuit board (PCB) substrate with parallel electrodes distributed across its surface. This PCB is made primarily of polyimide, which has been shown to require precursor diffusion before regular ALD can commence^33,34^.

Zinc oxide films were deposited on the PCB substrates and the resistances at all locations on the substrates were measured throughout the depositions. This enabled real-time monitoring of the spatial uniformity of the growing film. We were able to correlate specific resistance values with areas with visible spatial inhomogeneity. It also enabled real-time observation of different growth modes. It was discovered that the resistance of the films is very high until a critical amount of material has been deposited. Atomic force microscopy (AFM) and scanning electron microscope (SEM) measurements suggest that this change in resistance is due to the nucleation of small “islands” of zinc oxide, which gradually connect into a cohesive film. Furthermore, monitoring the film resistance during post-deposition cooling enabled immediate identification of metallic or semiconducting behaviour within the conductive ZnO films.

## Methods

**Figure 1:**
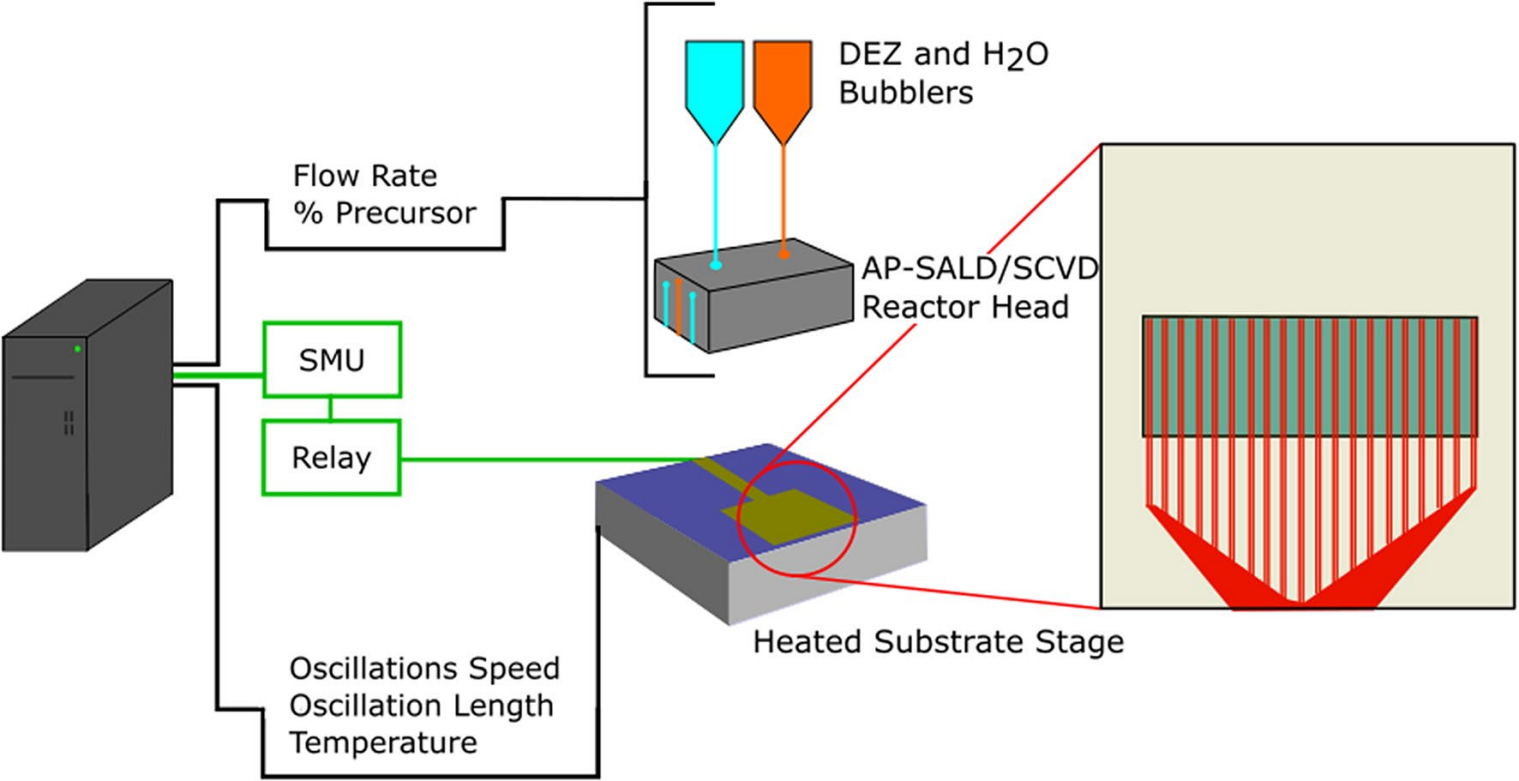
In-situ electrical measurement system. A flexible PCB substrate is placed on the oscillating substrate stage of an AP-SALD/CVD system and electrical resistance is measured at 20 trace pairs, shown in red, across the substrate throughout the deposition of a thin film. Most of the trace is covered by polyimide, the exposed area is shown in blue, dimensions of the PCB substrate can be found in Fig. S1.

Figure 1 illustrates our novel in-situ electrical measurement system. The PCB substrates were placed on the heated, oscillating substrate stage of a custom-built close-proximity AP-SALD/CVD system and held in place with both polyimide tape and with vacuum suction. The PCBs are made of polyimide, with twenty pairs of 0.12 mm diameter gold-coated copper traces (each pair is shown in Fig. 1 in red). A 15 mm length of each trace (area shown in blue) is exposed to the AP-SALD/CVD process; the remaining area is masked by an additional layer of polyimide. The exact dimensions of the PCB can be found in Fig. S1 of the Supplementary Information. The resistance for each trace pair was measured after every five oscillations of the substrate stage using a Keysight B2901A source measurement unit (SMU). The resistance for each trace pair was measured 100 times, and the average of all measurements was recorded after discarding any outliers. All films showed the presence of a Schottky barrier upon measuring the current–voltage response, as expected for a ZnO–Au junction, but the measurement voltage (5 V) was higher than the barrier height in each case.

The close-proximity AP-SALD/CVD system functions by oscillating a heated substrate below a reactor head consisting of parallel gas channels 5 cm long that contain alternating flows of precursor vapours and nitrogen, as detailed in previous reports^30,35^. ZnO was deposited using alternating flows of diethylzinc (DEZ) and water, each produced by bubbling nitrogen through them at a rate of 150 SCCM. As can be seen in Fig. 1, the bubbled precursor flows are further diluted with pure nitrogen at a flow ratio of 15:85 (bubbled precursor flow:pure N_2_ flow). Two water and one DEZ flows were directed onto our specialized substrate, which was heated to a temperature of 100 °C, 125 °C, 150 °C, or 175 °C. The substrate stage is comprised of nickel-plated copper and due to its high thermal conductivity maintains a consistent temperature across the substrate. Most of the excess precursor under the reactor is removed by vacuum-assisted exhaust channels on the reactor (not shown in Fig. 1), and the remainder is safely disposed of by the fume hood enclosure. The PCBs were found to be suitable for deposition temperatures below 180 °C. Above 180 °C, a finite resistance could be measured in the bare PCBs. 500 oscillations were performed for each deposition. A reactor-substrate distance of 100 ± 10 µm was maintained for all depositions. For a small reactor-substrate spacing and suitable flow rates, the alternating precursor flows can be isolated from one another, such that self-limiting reactions occur on the surface of the substrate, resulting in atomic layer-by-layer growth, in a manner analogous to conventional ALD^5,21,35–37^. For large substrate-reactor spacings, the precursor gases can mix and react in the gas phase, resulting in CVD and higher growth rates. Based on previous studies of ZnO with a similar AP-SALD/CVD system^36,38^, CVD is expected for the deposition conditions used here.

An Auriga 40 FIB/SEM system with a 2 kV electron beam was used to image the film surfaces and to estimate the final film thicknesses. AFM measurements were taken with a Dimension 3100 scanning probe microscope in tapping mode and analyzed using Gwyddion. Grazing incidence X-ray diffraction (GIXRD) measurements were performed with a PANalytical X'pert Pro MRD HR-XRD, with an incident wavelength of 1.54 Å. Photoluminescence measurements were taken with an excitation wavelength of 300 nm, with a 4.5 mm aperture on a Horiba QuantaMaster 8000.

## Results

**Figure 2:**
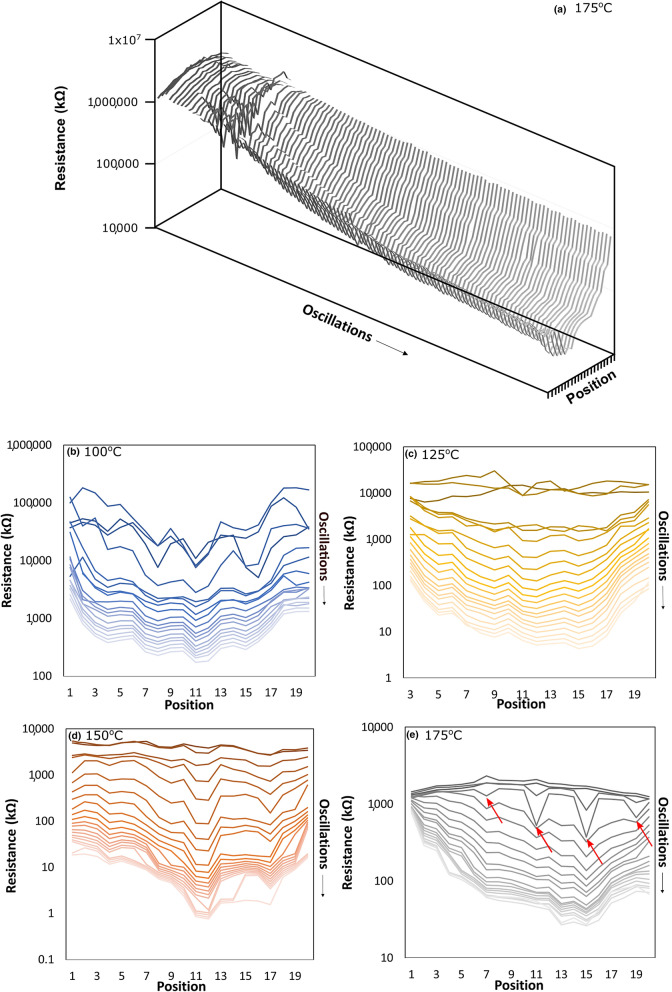
(**a**) All resistances measured throughout the deposition of a ZnO film on the polymer substrate at 175 °C. A selection of resistances measured across the polymer substrate during the deposition of a ZnO film at (**b**) 100 °C, (**c**) 125 °C, (**d**) 150 °C, and (**e**) 175 °C (arrows indicate locations with a temporarily increased deposition rate).

This in-situ electrical characterization technique generated a large amount of quantitative data on the resistance of ZnO films grown on the flexible substrates. Figure 2a shows all resistances measured at different trace pair positions on the polymer substrate when depositing a ZnO film at 175 °C. Plots for the other deposition temperatures (100 °C, 125 °C, 150 °C) are shown in Fig. S2 of the Supplementary Information and are similar in appearance. As expected, the resistances are initially high then decrease as the film is deposited and its thickness increases. Figure 2b–e show the resistances measured on the PCBs for the different substrate temperatures with only every tenth measurement shown for clarity. In the case of the deposition at 125 °C, the first trace pair was partially obscured by the tape used to attach the PCB to the stage, and so was omitted. For all temperatures, the measured resistance is higher at the outer positions of the polymer substrate (1 and 20) and lowest in the central area of the substrate. The outer substrate positions are located underneath the ends of the reactor head where the precursor gases are more likely to escape out the sides of the reactor into the fume hood. Thus, the higher and lower resistances observed at the edges and centres of the films are largely attributed to smaller and larger film thicknesses, respectively. This was confirmed by visual inspection of the films, as shown in Fig. S3, where variations in film colour indicate variations in thickness. The variation in thickness across the film surface was also observed by SEM. In addition, the outer substrate positions are likely to be exposed to more oxygen from the surrounding atmosphere, whereas the central portion of the substrate remains better isolated from the external environment. It has recently been reported that oxygen exposure limits the mobility and hence conductivity of ZnO-based films deposited by open-air methods via barrier formation at the grain boundaries, such that this may also contribute to the higher resistances observed at the outer substrate positions^39,40^. The ZnO photoluminescence spectra at different positions on the substrate can be seen in Fig. S4, where the green (495–570 nm) photoluminescent peak is notably larger at the outer substrate position, despite the film being thinner at this location. This peak, here observed at around 505 nm, is commonly attributed to zinc vacancies/oxygen interstitials^41^ and so the higher photoluminescence peak on the edge of the film suggests a higher relative concentration of oxygen, consistent with the higher resistance. This in-situ measurement technique therefore provides real-time monitoring of the spatial uniformity of the growing film, where higher resistances correspond to regions with lower growth rates and film thicknesses, and potentially slightly different stoichiometries.

This in turn means that the spatial uniformity of films can be monitored during growth, and deposition parameters can be changed in real time to correct for abnormally high or low growth rates or any non-ideal deposition conditions. For example, it can be seen in Fig. 2e that four of the trace pairs (7, 11, 15, and 19, highlighted with arrows) show very rapid decreases in resistance (suggesting a significant increase in thickness) at an early stage, inconsistent with the rest of the film. This appears to have occurred because these traces were located over the suction holes on the substrate stage used to secure the substrate to the stage. These regions of the flexible polymer substrate were pulled downwards by the suction, creating larger reactor-substrate spacings, which resulted in more mixing of the precursor gases and more CVD with a larger growth rate (visible in Fig. S3). The vacuum was subsequently turned off during the deposition and the film surface became more uniform.

**Figure 3:**
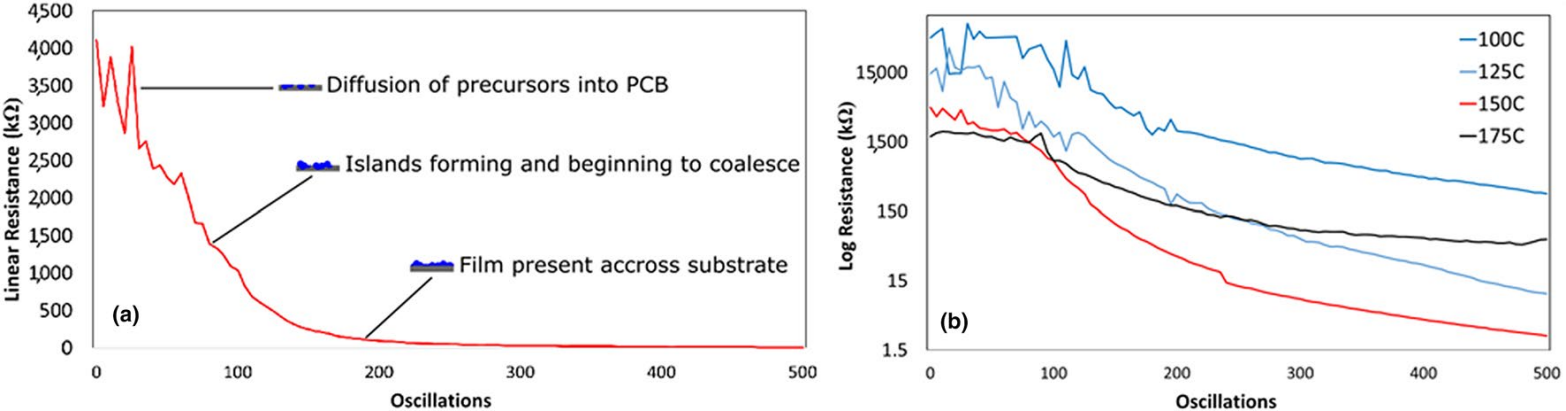
Change in resistance over time at the centre of the film for depositions at (**a**) 150 °C on a linear plot and (**b**) all deposition temperatures on a log-linear plot. Three distinct growth regions are highlighted in (**a**).

It is seen in Fig. 2, as well as in Fig. S2 of the Supplementary Information, that the resistance measurements taken during the initial stages of film deposition are noisier and become more uniform as the film becomes thicker. This is particularly pronounced in the low-temperature films. To gain insight into the film nucleation and growth, the temporal variation in resistance was analyzed. Figure 3a shows the resistance measured in the centre (location 10) of the film produced at 150 °C, throughout the 500 oscillation deposition. Three distinct regions can be seen. In the first region, the measured resistance is large and noisy. As noted earlier, polymers tend to be permeable and can have few nucleation sites, such that ALD precursors permeate within the film and accumulate until sufficiently high concentrations enable film nucleation. It is expected that this first region corresponds to the diffusion of the diethylzinc and water precursors into the polymer substrate and the formation of isolated nucleation sites (islands). After approximately 80 oscillations, the measured resistance becomes stable and drops precipitously from more than a megaohm to a few kilo-ohms. This behaviour is unlike Frank–van der Merwe growth that characterizes ALD, where the thickness would increase linearly, as each oscillation would add a complete monolayer of ZnO^42^. The observed variation in resistance is more consistent with Volmer-Weber growth, which is observed during CVD and is characterized by the formation and coalescence of isolated islands and not epitaxial growth^43^. This suggests that the second region of Fig. 3a corresponds to the continued formation and growth of zinc oxide islands, which gradually coalesce into a continuous film, which would produce a lower and more stable resistance measurement. In the third region, the resistance approaches a linear dependence on the number of oscillations. A linear decrease in the resistance is expected when the islands are completely connected, and the resistance simply changes as the film thickness increases. The formation of continuous, polycrystalline ZnO films on the flexible substrates was confirmed by SEM and GIXRD in Figs. S5 and S6 of the Supplementary Information respectively. Examination of the (002) ZnO GIXRD peaks in Fig. S7 of the Supplementary Information indicates that the crystallite size increases with temperature from approximately 139 nm in the film deposited at 100 °C to 159 nm in the film deposited at 175 °C, consistent with the larger grains observed in the SEM images in Fig. S5. These three distinct growth regions were also observed for the films deposited at temperatures of 100 °C, 125 °C, and 175 °C (Fig. S8 of the Supplementary Information).

The temporal evolution of the resistance for the four deposition temperatures is compared in Fig. 3b on a semi-log plot. Again, the measurement from the centre position on the polymer substrate is shown. It is evident that the resistance in the early stages of the deposition gets progressively smoother as the substrate temperature is increased from 100 to 175 °C. This suggests that faster island formation and initial coalescence (i.e. an accelerated region 1) occurs at elevated substrate temperatures. Since the diffusion and nucleation behaviour of ALD precursors on polymers is highly temperature dependent^32^, it is likely that the higher temperature enables chemisorption of the diethylzinc onto the surface of the polyimide directly, or that sufficient diffusion and reaction happens much earlier than at lower deposition temperatures. This is consistent with the SEM images presented in Fig. S5 of the Supplementary Information, where the grains in the ZnO film deposited at 100 °C are smaller and more irregular compared to the grains in the films deposited at higher temperatures, which appear more clearly formed with a regular structure. For the third growth region, it is seen that the resistance becomes most linear for the film deposited at 175 °C, whereas at lower temperatures, the resistance evolution maintains a stronger non-linear component at later stages of the deposition. Given that continuous films were observed in the SEM images in Fig. S5, and that a relatively constant deposition rate is expected at the later stages, this suggests that film properties other than thickness may be varying throughout the deposition. At 175 °C for example, the organic ligands remaining from the diethyl zinc may be removed quickly from the films, whereas at lower temperatures this removal may be slower and more gradual, resulting in a continued non-linear decrease in resistance throughout the deposition period. While detailed chemical analysis would be required to verify changes in the film properties, these in-situ measurements provide preliminary evidence of evolving material properties during the deposition.

**Figure 4:**
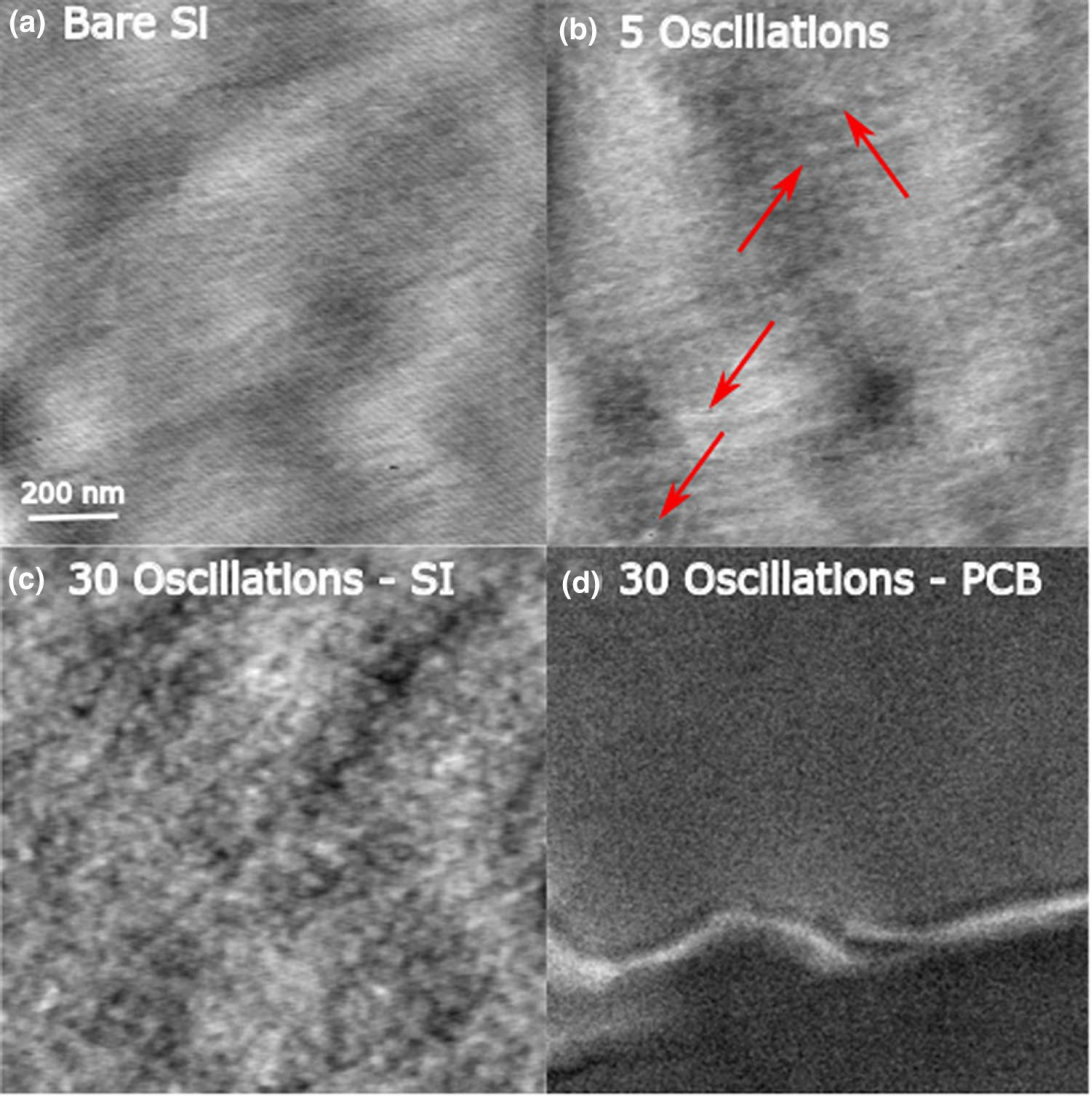
Images of (**a**) a bare silicon wafer (AFM), (**b**) silicon wafer with 5 oscillations of ZnO deposition at 150 °C (AFM), (**c**) silicon wafer with 30 oscillations of ZnO deposition at 150 °C (AFM) and (**d**) polyimide with 30 oscillations of ZnO deposition at 150 °C (SEM). Island formation and coalescence is observed, as well as delayed island growth on polymer substrate.

Reliable AFM measurements could not be performed on the flexible polymer substrates. However, AFM analysis of ZnO films deposited on silicon substrates under identical conditions at 150 °C confirmed the formation of ZnO islands, consistent with the proposed Volmer–Weber growth. Figure 4 shows AFM images for (a) a bare Si substrate, as well as a Si substrate after (b) 5 and (c) 30 AP-SALD/CVD oscillations. After 5 oscillations, a few distinct islands of ZnO (highlighted by arrows) can be seen against the background of the Si substrate. After 30 oscillations, many ZnO islands are evident on the Si, and it appears that the islands have already begun to grow together into a polycrystalline film. This apparent coalescence of ZnO islands on silicon after 30 deposition oscillations is in contrast to the resistance trend observed in Fig. 3a, where approximately 80 oscillations were required before the resistance stabilized and fell, indicating the formation of a more continuous film on the polymer substrate via the coalescence of ZnO islands. This is attributable to the fact that on the polymer substrate, it is expected that the island growth would be delayed until suitable nucleation sites had been formed by the diffusion of the precursor into the substrate, as discussed earlier. This was confirmed by SEM analysis of a polyimide substrate after 30 AP-SALD/CVD oscillations at 150 °C (Fig. 4d). After 30 oscillations, no ZnO islands can be distinguished from the background texture of the polyimide substrate, indicating the precursor diffusion and film nucleation is still at an early stage. These results are also consistent with our prior report on the temperature-dependent nucleation of ZnO using our AP-CVD system on non-porous substrates. Using in-situ reflectance spectrometry we saw that the films coalesced more quickly when deposited at higher temperatures even on borosilicate glass. This is due to higher oxygen and hydroxyl content in the atmosphere, compared to conventional vacuum ALD, promoting reactions while the precursors are still in the vapour phase^44–46^.

AP-CVD growth rates have been reported to be constant throughout the deposition, on various substrates^47,48^. Thus, by measuring the final film thicknesses, we can determine the approximate growth rates. The film thickness measured on the polyimide PCB substrate using SEM for each deposition temperature is shown in Table 1, along with the corresponding growth rate. The growth rates increase from approximately 0.48 nm/cycle at 125 °C to 1.77 nm/cycle at 175 °C (one AP-SALD/CVD oscillation corresponds to 2 precursor exposure cycles). These vary from the reported growth rate of 1.8 Angstrom/cycle for ALD of ZnO^23^, confirming that CVD is occurring on our samples, as expected. The increase in growth rate with temperature is consistent with CVD, and is also consistent with the electrical measurements in Fig. 3b that indicated accelerated ZnO nucleation and island coalescence at higher deposition temperatures.

**Table 1:** Measured thicknesses at the centre of the film and estimated growth rates for the films grown at each of the four temperatures.

Deposition temperature (°C)	Film thickness (nm)	Growth rate (nm/cycle)
100	560	0.56
125	480	0.48
150	1560	1.56
175	1770	1.77

A relatively high final resistance is noted for the film deposited at 175 °C in Fig. 3b, despite it having the largest final film thickness in Table 1. Oxygen vacancies are one proposed source of charge carriers in ZnO^49,50^. It has previously been shown that annealing ZnO films in air results in a reduction in the concentration of oxygen vacancies, or an increase in oxygen interstitial trap states, and hence a decrease in the available charge carriers^51^. As noted earlier, it has also been reported that oxygen tends to accumulate at the grain boundaries of ZnO-based films, slowing transport of charges from one grain to another as the electrons must tunnel through this barrier^39,40^. This effect leads to an increase in film resistance of a couple orders of magnitude, and is particularly noted in AP-SALD films. Therefore, films deposited in air at higher temperatures would be expected to have fewer carriers, more trap states, and higher resistivities. The higher final resistance observed at 100 °C versus 125 °C in Fig. 3b despite their similar thicknesses may be attributable to a reduced crystallinity (and hence carrier mobility) for the film deposited at 100 °C^52,53^, consistent with the small, irregular grains observed by SEM (Fig. S5) and smaller crystallite size observed by GIXRD (Figs. S6, S7).

**Figure 5:**
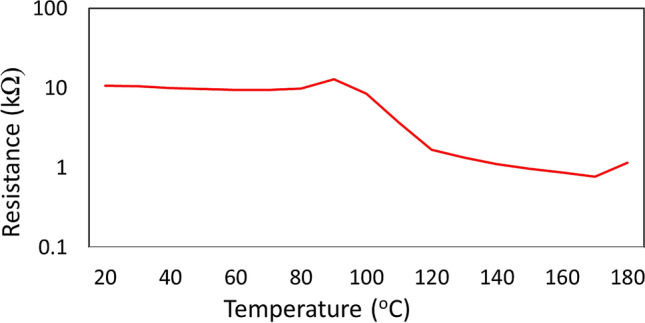
Change in resistance of trace pair 10 from the 150 °C film as it is heated on a hot plate from room temperature to 180 °C.

**Figure 6:**
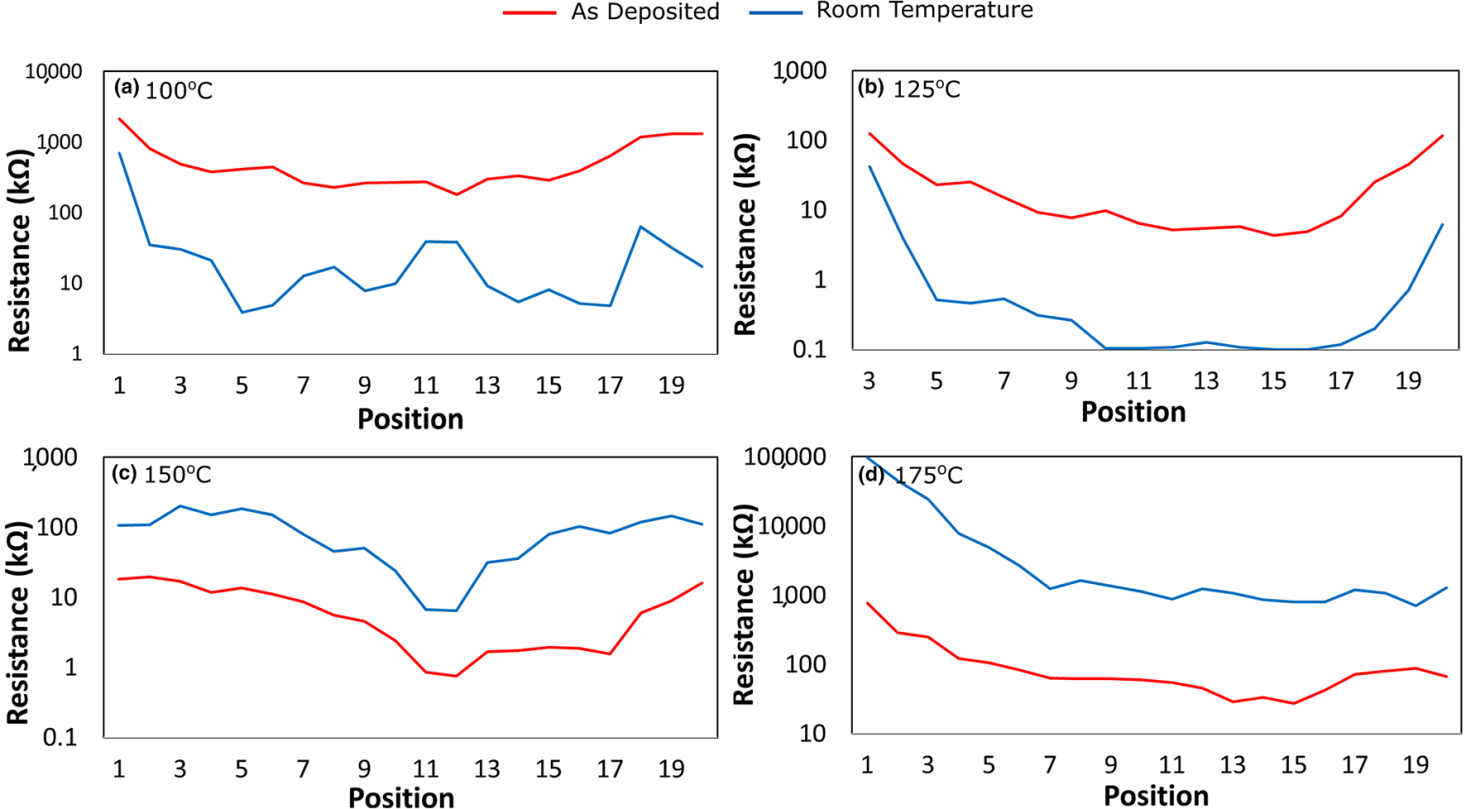
Resistance across the PCB’s measured as deposited and at room temperature, films deposited at (**a**) 100 °C, (**b**) 125 °C, (**c**) 150 °C, and (**d**) 175 °C.

Figure 5 shows the resistance at a central location on the film deposited at 150 °C as the film was heated from room temperature up to 180 °C. The resistivity of ZnO is highly temperature-dependent^53^, and the PCBs developed in this work enable monitoring of the resistance across the film surface during cooling to room temperature, as well as during subsequent annealing. Figure 6 shows the resistance measured across the film surface at the end of the deposition (red curve), as well as after cooling to room temperature (blue curve), for the four different deposition temperatures.

It is seen that for the films deposited at 150 °C and 175 °C, the resistance at all locations increases as the films are cooled to room temperature, consistent with semiconductor behaviour. In contrast, the resistance of the films deposited at lower temperatures of 100 °C and 125 °C decreased when they were cooled to room temperature, consistent with a more metallic or degenerate behaviour.

## Discussion

We developed a novel in-situ method for observing the nucleation, growth, and electrical properties of AP-CVD films grown on polymer substrates. Unlike conventional four-probe resistivity measurements, our method can resolve the changes in resistance over both time and space, providing more information. This is particularly applicable to the field of flexible electronics, for wearable and wireless devices, as the initial diffusion of the ALD precursors into polymer substrates is highly temperature-dependent, and a full understanding of this mechanism will result in more consistent thin film depositions. Of note is the demonstration of very high measured resistances for many more cycles than would be expected on conventional rigid substrates, due to this diffusion. We also found that films deposited at higher temperatures became more resistive when measured at room temperature, due to the atmospheric deposition also functioning as an annealing step. As shown in Fig. 2a, this technique also allows for comprehensive data sets to be produced from a small number of depositions, which will be valuable for machine learning applications to materials science. By using machine learning to understand the interrelations between deposition conditions such as substrate temperature, and film properties, AP-CVD films can be tailored to specific applications. This will lead to more efficient devices which can be constructed with a low-cost and high-throughput technique. Combining this technique with in-situ thickness measurements in the future would allow the resistivity of the film to be monitored in real-time (for ohmic contacts), also allowing for greater control of final film properties before being used in a device.

This system is limited in that it can only spatially resolve the resistance in one direction, when the thickness can vary across the film, leading to some uncertainty. Here the nucleation of ZnO was studied. ZnO is a technologically important transparent semiconductor that is being applied in various flexible devices, such as wearable devices and photovoltaics. Diffusion behaviour is highly dependent on the chemical interactions between the precursor and substrate, and so additional studies will need to be done with other materials. However, our method is applicable to a wide range of possible metal chalcogen films which can be used in flexible electronic devices.

## Supplementary information

Supplementary Information.
