# Supplementary material for: In-situ spatial and temporal electrical characterization of ZnO thin films deposited by atmospheric pressure chemical vapour deposition on flexible polymer substrates
Source: Sci Rep. 2020 Nov 17;10:19947. doi: 10.1038/s41598-020-76993-4 (PMC7672091; doi:10.1038/s41598-020-76993-4)
Supplement: Supplementary file 1 — Supplementary Information. [file 41598_2020_76993_MOESM1_ESM.pdf]

## Supplementary Information

## In-Situ Spatial and Temporal Electrical Characterization of ZnO Thin Films Deposited by Atmospheric Pressure Spatial Chemical Vapour Deposition on Flexible Polymer Substrates

*Alexander Jones, Kissan Mistry, Manfred Kao, Ahmed Shahin, Mustafa Yavuz, Kevin P. Musselman**

*kevin.musselman@uwaterloo.ca

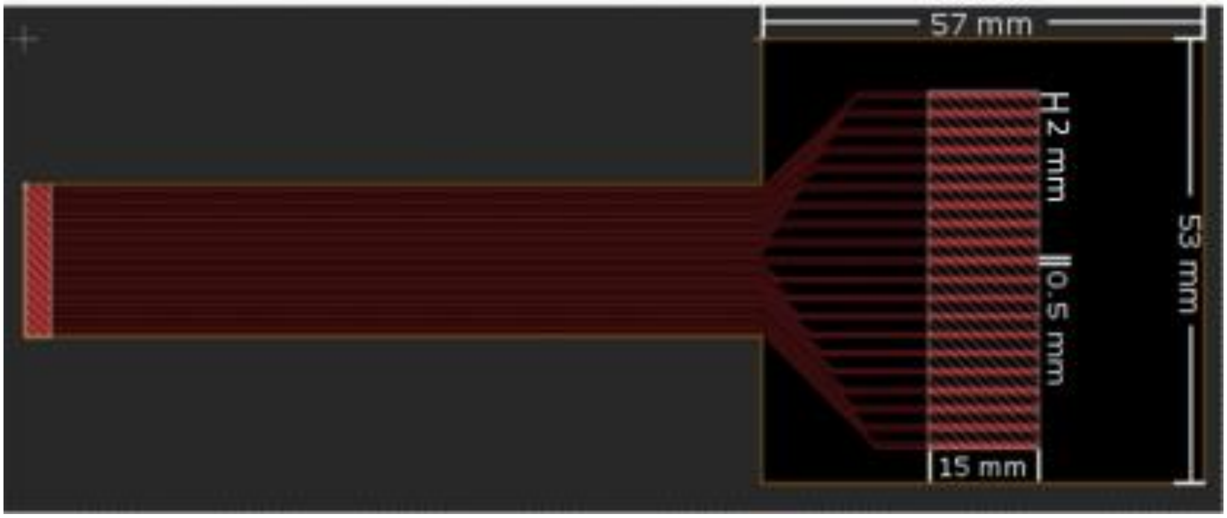


*Figure S1: Labelled schematic of the PCB used in these experiments*

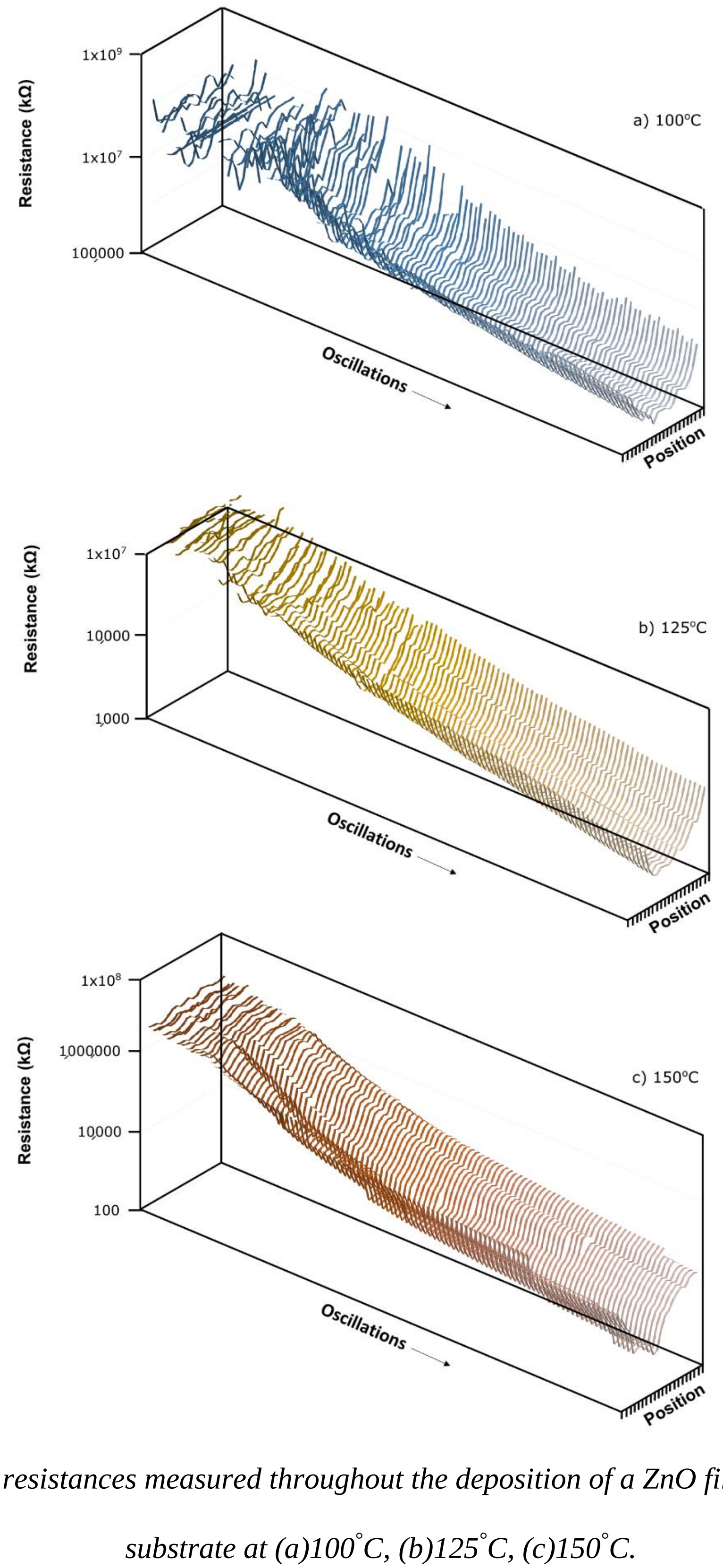


*Figure S2: All resistances measured throughout the deposition of a ZnO film on the polymer substrate at (a)100˚C, (b)125˚C, (c)150˚C.*

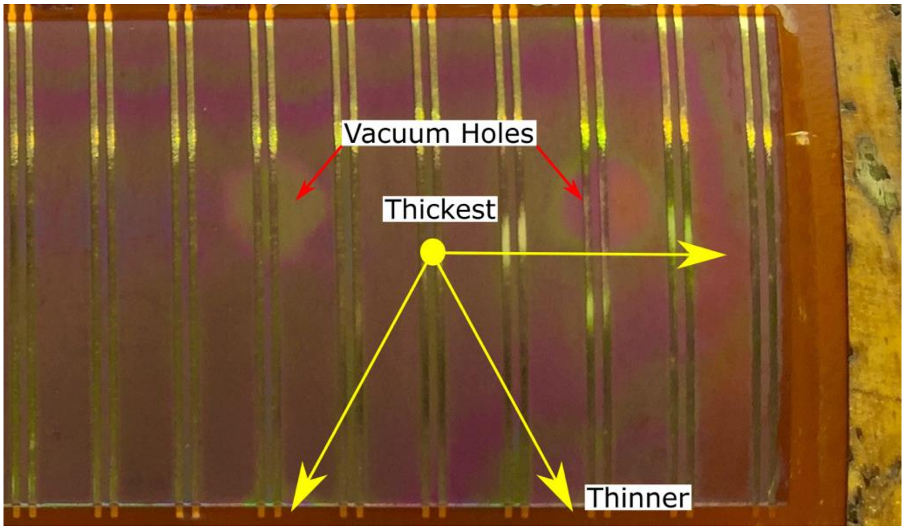


*Figure S3: Close-up image of the film produced at 175$^{o}$C showing the variations in film colouration (due to thickness variation) towards the outer parts of the deposition area and where the stage vacuum deformed the PCB.*

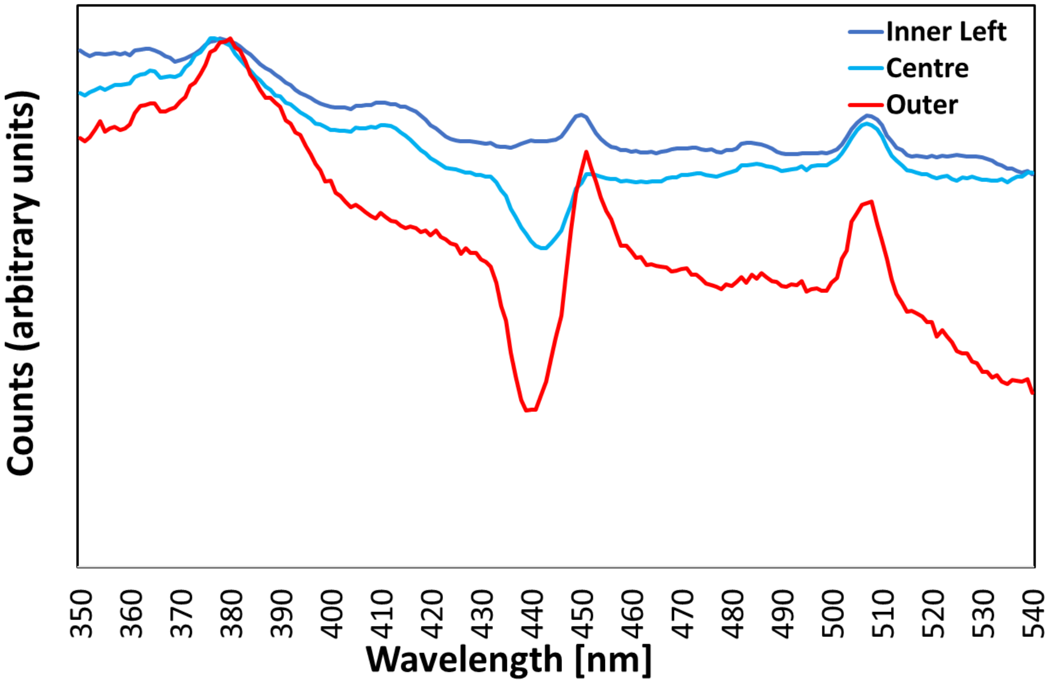


*Figure S4: Photoluminescence peaks measured at different locations on the 175°C film. Suppressed green (475-570nm) peaks in the measurements taken at central and inner left film positions indicate lower oxygen concentration relative to the outer edge.*

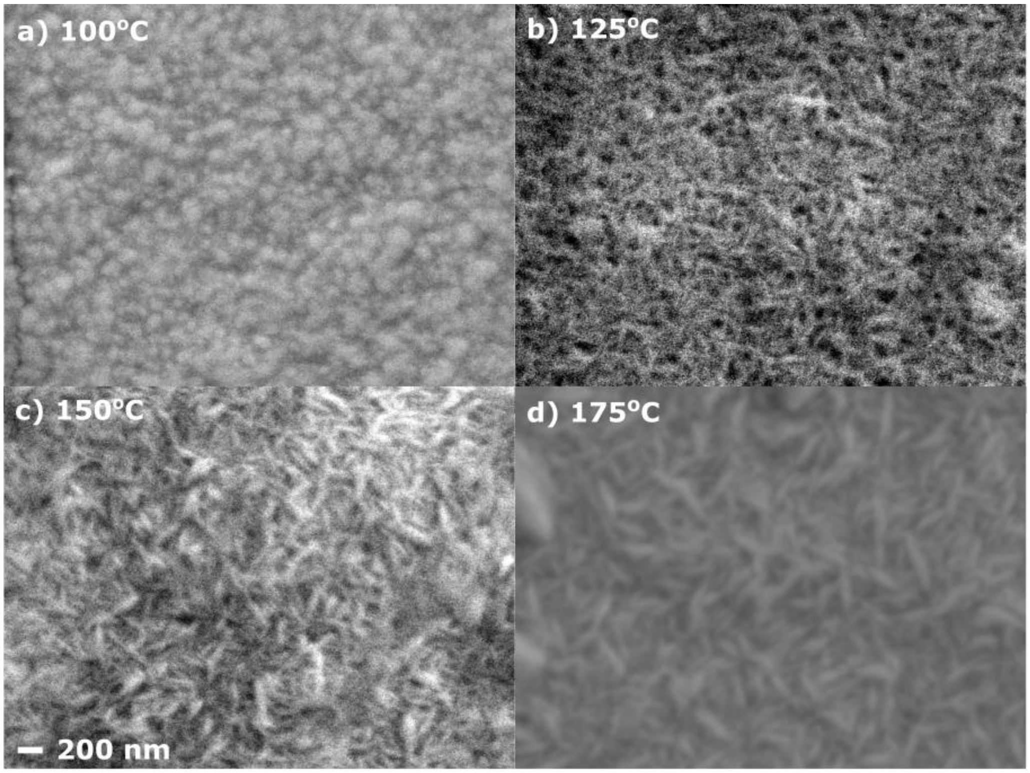


*Figure S5: – SEM images of 500 oscillation films deposited on the PCB at (a) 100°C, (b) 125°C, (c) 150°C, and d) 175°C. Changes in crystal shape and size can be observed, image taken with 5kV beam and 30,000x magnification.*

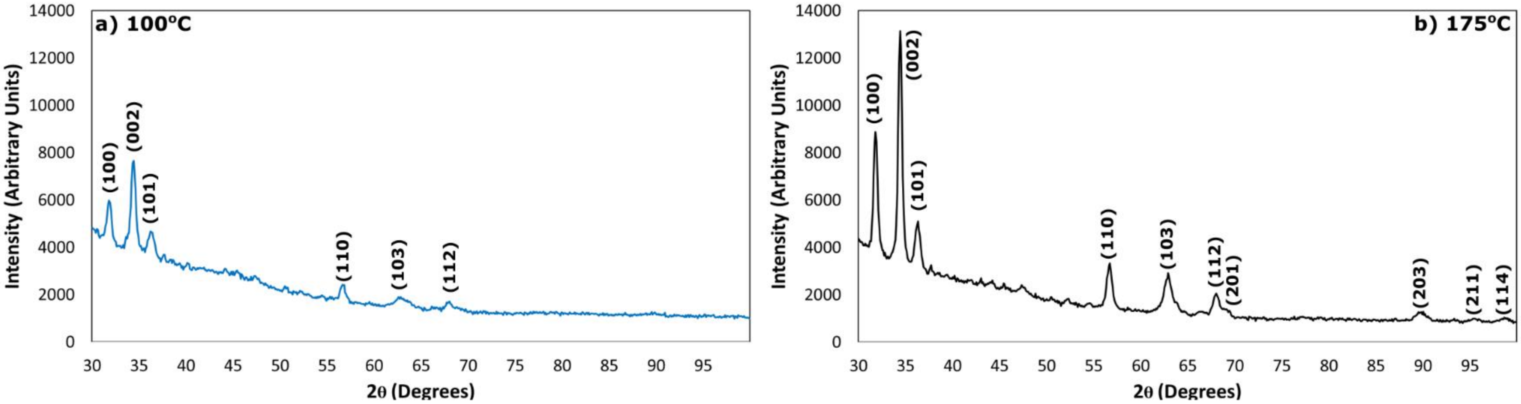


*Figure S6: GIXRD measurements for films produced at a) 100°C and b) 175°C. Peaks were identified using the ZnO reference data card 00-036-1451.*

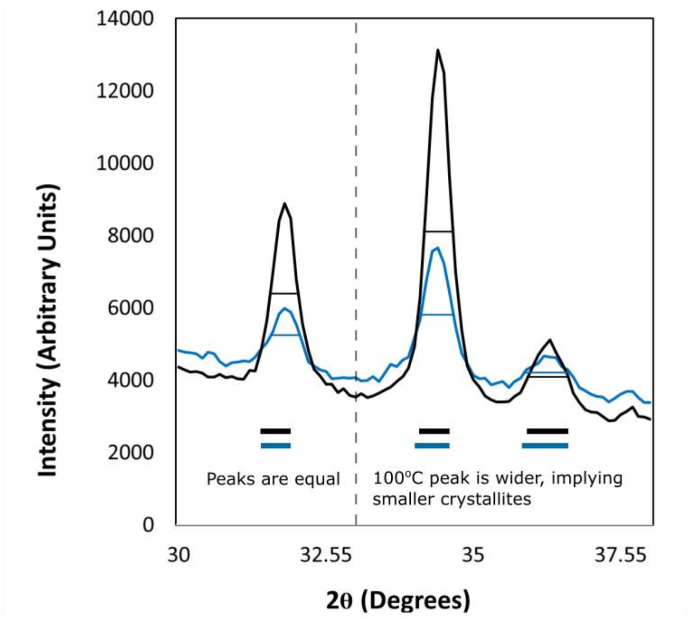


*Figure S7: The first three GIXRD peaks from the films produced at 100°C and 175°C. Estimated crystallite sizes based on the Scherrer equation at the (002) peak are 138.5 nm at 100°C and 159 nm at 175°C.*

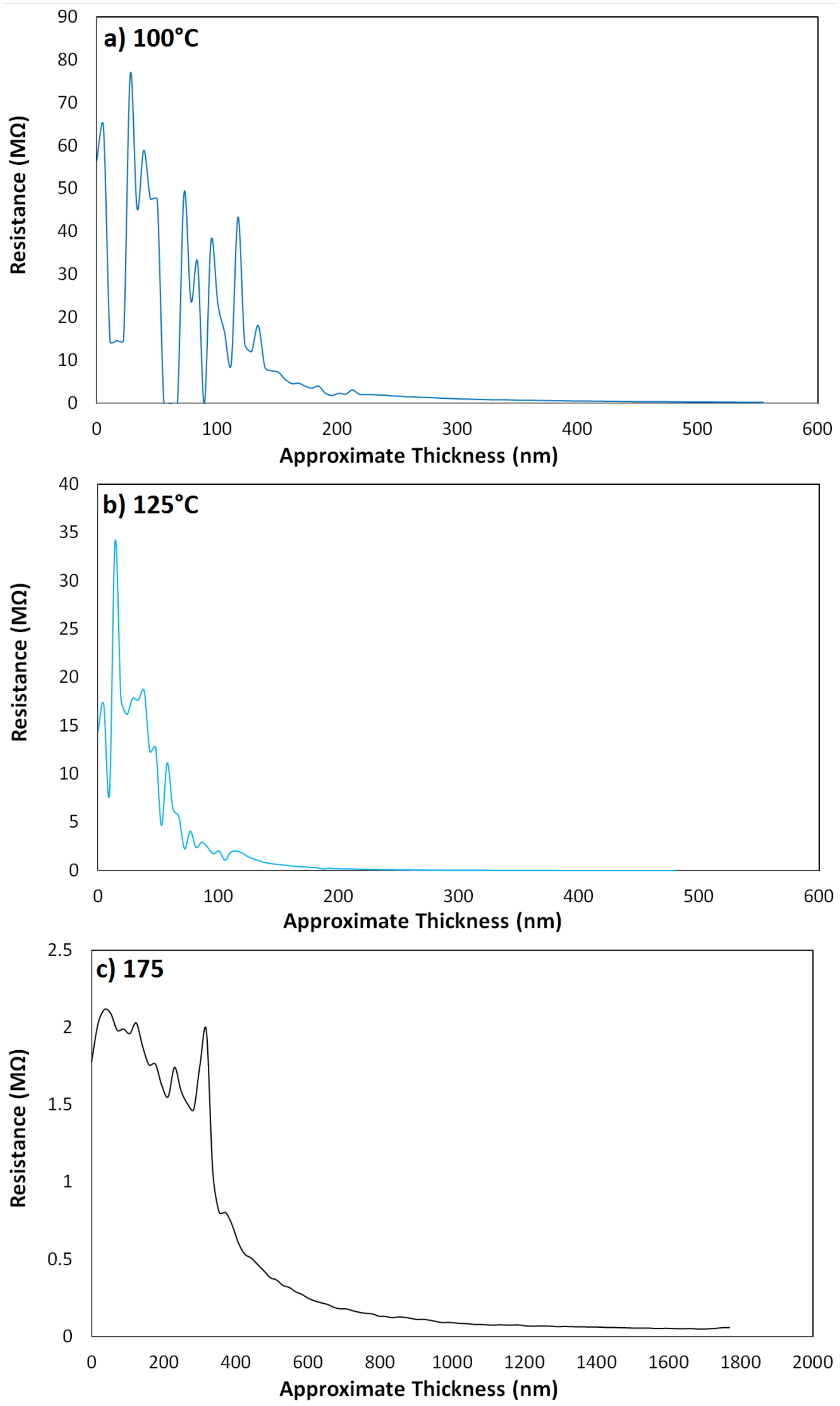


*Figure S8: Resistance vs. approximate thickness measured at trace pair 10 for films deposited at a) 100$^o$C, b) 125$^o$C, and c) 175$^o$C*
